# Development of advantus™(imidacloprid) soft chewable tablets for the treatment of *Ctenocephalides felis* infestations on dogs

**DOI:** 10.1186/s13071-015-1020-1

**Published:** 2015-08-04

**Authors:** Tariq Qureshi, William R. Everett, Kathleen G. Palma

**Affiliations:** Bayer HealthCare, LLC, Animal Health Division, Shawnee Mission, KS 66201-0390 USA; BerTek, Inc., Greenbrier, AR 72058 USA; Piedmont Animal Health LLC, Greensboro, NC 27410 USA

**Keywords:** Imidacloprid, Advantus, Oral, Soft chews, *Ctenocephalides felis*, Fleas, Canine

## Abstract

**Background:**

Studies reported here were conducted to evaluate the safety and effectiveness of advantus™ (imidacloprid) soft chewable tablets for the treatment of flea (*Ctenocephalides felis*) infestations on dogs and puppies 10 weeks of age or older and weighing 4 pounds or greater.

**Methods:**

A pharmacokinetic study was conducted to evaluate parameters of orally administered imidacloprid. A dose confirmation study was conducted to confirm the efficacy of 0.75 mg/kg at 8, 12 or 24 hours post-treatment. A knockdown and speed of kill study was conducted to confirm the efficacy of 0.75 mg/kg dose at 0.5, 1, 4 or 24 hours post-treatment. The safety of a daily dose administered for six months at approximately 1, 3, and 5 times the maximum exposure dose of 3.75 mg/kg was evaluated in puppies. A field study was conducted to evaluate the safety and efficacy of a daily oral dose of 0.75 mg/kg for 14 days in client-owned dogs.

**Results:**

The pharmacokinetic parameters of the final imidacloprid oral formulation were; T_max_ 1.31 hours, C_max_ 690.0 ng/mL, AUC 2615.5 h*ng/mL and half-life was 2.2 hours.

The efficacy of 0.75 mg/kg BW was 98.6 %, 99.9 % and 100 % at 8, 12 and 24 hours post-treatment, respectively. The live flea counts were significantly different (*p* < 0.0001) and the treatment was well tolerated.

The flea counts at 1 hour post-treatment were significantly lower in the treated group and the speed of kill efficacy was 96.6 % at 4 hours post-treatment in the knockdown and speed of kill study.

The target animal safety study showed that the advantus™ soft chewable tablets administered orally to 10-week-old puppies once daily for 6 months at approximately 1, 3 and 5 times the maximum dose of 3.75 mg/kg was well tolerated and did not produce clinically relevant findings in Beagles.

In the field study, efficacy of the soft chewable tablets administered daily for 14 days to flea-infested dogs was 98.2 %.

**Conclusion:**

Imidacloprid administered orally as a soft chewable tablet for the treatment of fleas on dogs was safe and highly effective with a rapid knockdown effect and rapid elimination.

## Background

The cat flea, *Ctenocephalides felis*, is the most important parasite found on dogs worldwide [1]. Flea infestations result in itching, anemia in heavy infestations, and flea allergy dermatitis (FAD). Fleas are also competent vectors of several pathogens, including bartonellosis, rickettsial infections [2, 3] and *Dipylidium caninum* [4]. Significant advancements have been made within the past 20 years for the control of flea infestations of dogs and cats. Control methods have evolved from treating the pet and its indoor and outdoor environment to only treating the pet [5] because active ingredients have been developed that have rapid activity, persistent activity, and in some cases broad spectrum of activity. In addition to being highly efficacious, various delivery systems have also been developed to provide convenience of application including sprays, topical spot-on, collars and systemic products, such as injectable or oral formulations. These advancements have made it possible to effectively control flea infestations on pets at a very high level.

Imidacloprid is a neo-nicotinoid insecticide introduced in the USA in 1996 as a veterinary and agricultural product [6]. Imidacloprid is a competitive inhibitor of the nicotinic acetylcholine receptors of target insects in postsynaptic nerves which prevents acetylcholine from binding and transmitting information resulting in incoordination, loss of motor skills and death of the insect [7, 8]. The compound is effective against insects because they have a large proportion of nicotinic acetylcholine receptors and because imidacloprid has a higher binding affinity to insect nerve receptors than to mammalian receptors [9]. As with other neo-nicotinoids, imidacloprid has selective activity against fleas and other insect species with less activity against acarines [10].

In animal health, imidacloprid was first developed as a topical insecticide (Advantage®) with excellent efficacy for treatment and control of fleas on dogs and cats. Single spot-on application of imidacloprid at 7.5 and 10.0 mg/kg killed fleas on dogs within 24 hours and had effective residual activity for 34 days [6]. The efficacy was not affected by shampooing or exposing the dog to water [11, 12]. Combining imidacloprid with other products extended the spectrum of activity. Imidacloprid was combined with permethrin (K9 Advantix®, Advantix®) to extend activity against ticks [13, 14] and add repellent activity against sand flies on dogs [15]. The combination of imidacloprid and moxidectin (Advantage Multi®, Advocate®) is used for prevention of heartworm disease [16] and for treatment of fleas, intestinal nematodes [17], sarcoptic mange [18], demodex mange [19] and ear mites [20]. Imidacloprid combined with pyriproxyfen (Advantage II®) extended the activity against all life stages of fleas [21].

This manuscript describes five studies conducted for the development of advantus™ soft chewable tablets containing imidacloprid for treatment of flea infestations on dogs and puppies 10 weeks of age or older and weighing 4 lb (1.8 kg) or greater. The advantus™ soft chewable tablets are available in two sizes; 7.5 mg imidacloprid for dogs weighing 4 to 22 lb (1.8–10 kg) in weight and 37.5 mg imidacloprid for dogs weighing 23 to 110 lb (10–50 kg) in weight. Studies were conducted to establish the pharmacokinetic characteristics, for substantial evidence of activity against fleas (efficacy and speed of kill), for confirmation of safety in target species, and to evaluate field experience regarding safety and efficacy of the product as used by pet owners.

## Methods

### Ethical approval

All laboratory protocols were approved by the Institutional Animal Care and Use Committees at each facility. Animal husbandry and housing were in compliance with Animal Welfare Act Regulations. All owners who enrolled their dogs in the field study signed an Owner’s Consent form.

With the exception of the pharmacokinetic study, the studies reported here were conducted under Good Clinical Practice (GCP) and Good Laboratory Practice (GLP) standards in compliance with VICH GL9 guidelines June 2000, and 21CFR Part 511. The studies were designed in accordance with standard methods for evaluating efficacy of parasiticides for treatment of flea infestations [22] and as well as safety [23].

The protocols for the laboratory studies specified the use of purebred beagles or mixed-breed dogs that had not been treated with an acaricide, insecticide, or an insect growth regulator for the previous 60 days. All laboratory study dogs originated from test facility colony or were purchased from commercial sources and were acclimated to the test facility environment for 7 to 10 days prior to study initiation. Dogs were individually housed in indoor pens or cages that conformed to the national standards for area, size, lighting, and temperature. Water was available *ad libitum* and an adequate amount of commercial dog food was provided for daily maintenance.

During the acclimation period the dogs were bathed with a non-medicated shampoo and combed to remove any pre-existing fleas. Health observations were conducted daily during the conduct of studies. Pre-treatment physical examinations of all dogs were conducted. For the efficacy studies, a pre-treatment flea infestation followed 24 hours later with a flea comb count was conducted to determined susceptibility of the dogs to flea infestations. Pre-treatment flea counts were used to select the most susceptible dogs for randomization into study groups as defined by the protocol.

Fleas used for the laboratory efficacy studies were either purchased commercially or were reared at the site insectary as per site Standard Operating Procedures (SOPs). Vials containing approximately 100 recently emerged adult fleas were prepared for each dog for each designated infestation by vacuum system transfer. Each dog was infested by placing the fleas from each vial on the dorsal midline area and restraining the dog for a sufficient time to allow the fleas to move into the hair and establish infestation.

Fleas were counted using a fine toothed flea comb to recover fleas present in the animal’s fur. The combing procedure was as described in the site SOPs and conformed to industry standards. The total numbers of live and dead fleas collected were recorded on the data forms. Removed fleas showing no movements were considered dead.

The geometric mean numbers of live adult fleas on dogs in the control groups were compared to the geometric mean number of live adult fleas on treated dogs at each time point. Percent effectiveness was calculated using Abbott’s formula as follows:

$$ \mathrm{Efficacy}\ \left(\%\right) = 100\ \mathrm{X}\ \left(\mathrm{M}\mathrm{c}\ \hbox{--}\ \mathrm{M}\mathrm{t}\right)/\mathrm{M}\mathrm{c} $$

Mc = Geometric mean of live fleas in the control group

Mt = Geometric mean of live fleas in the treatment group

All analyses were performed using SAS/STAT® software (Version 9, SAS System for Windows, Copyright© 2002–2003 by SAS Institute Inc., Cary, NC, USA). An analysis of variance (ANOVA, SAS PROC MIXED) using log (live flea count +1) transformations compared the live flea counts of treatment group and the control at each time point. The comparisons were tested using the (two-sided) 5 % significance level. The fixed model analysis was used to analyze log-counts, with treatment group as a fixed effect.

### Pharmacokinetics

A pharmacokinetic study was conducted to determine the rate and extent of absorption of imidacloprid administered to eight dogs as a soft chewable tablet. The dogs were fasted overnight prior to dosing. Each dog was dosed orally with the final formulation of the soft chewable tablet containing 7.5 mg imidacloprid. Approximately 4 mL of blood was collected from each dog at pre-dose and at 0.25, 0.5, 1, 2, 4, 8 and 12 hours post-treatment. Serum was collected in 0.5 ml aliquot, frozen and sent to an analytical laboratory (En-CAS Analytical Laboratories, Winston-Salem, NC) for analysis using a validated analytical method. The limit of quantitation (LOQ) was 1.0 ppb.

Pharmacokinetic analysis was accomplished using WinNonLin (version 5.3, PharSight Corp., Inc. Mountain View, CA) to estimate individual pharmacokinetic parameters. Descriptive statistics (number of observations, means, standard deviations and minimum and maximum values) were determined for T_max_, C_max_ and AUC _last_.

### Dose confirmation

A GCP study was conducted to confirm the effectiveness of advantus™ soft chewable tablets (Investigational Veterinary Product (IVP)) administered at the minimum dose of 0.75 mg/kg body weight for the treatment of adult flea infestations on dogs.

Fifty-six dogs were acclimated at the test facility for 9 days prior to the study initiation and processed as previously described. Forty-eight dogs with the highest flea counts were selected for the study and randomized to one of three study groups of 16 dogs each for flea counts conducted at 8, 12 or 24 hours post-treatment. Within each study group, dogs were randomly assigned to one of two treatments groups; the advantus™ treated (IVP) group and the placebo treated (Control Veterinary Product, CVP) control group for a total of 8 dogs per treatment group. On Day −1, the body weight of each dog was measured for dose calculation and then all dogs were infested with approximately 100 recently emerged unfed adult fleas. The dogs were fasted overnight prior to dosing on Day 0. Dogs were randomly assigned to treatment order and treated on Day 0 with 7.5 mg imidacloprid IVP or placebo CVP soft chewable tablets, depending on their assigned treatment group. Comb counts were performed at 8, 12 and 24 hours post-treatment, respectively. General health observations were conducted daily from the start of acclimation through to the end of the study. Additional health observations for treatment related adverse reactions were conducted pre-treatment, and at 1, 2 and 4 hours post-treatment.

The geometric mean of live fleas (defined as alive or moribund) on dogs in the control groups were compared to the geometric mean of live fleas on treated dogs at each time point. Percent effectiveness was calculated and the transformed live flea count data was compared to determine the difference between the treated and the control groups at each time point.

### Knockdown and speed of kill

A GCP study was conducted to determine the speed of kill of the IVP administered orally at a minimum dose of 0.75 mg/kg body weight for the treatment of adult cat flea (*C. felis*) infestations of dogs.

Fifty-two dogs were acclimated to the test facility for 10 days. Forty-eight healthy dogs with the highest flea counts and meeting all other protocol requirements were enrolled in the study. The dogs were randomly assigned to two treatment groups either the sham treated control group (non-treated) or the IVP treated group. The two groups were randomized further to a combing time of 0.5, 1, 4 or 24 hours post-treatment .All dogs were weighed on Day −3 for accurate dosing. On Day −1, all dogs were infested with approximately 100 adult fleas. The dogs were fasted overnight prior to dosing on Day 0. All dogs were randomized to treatment order and those dogs assigned to the treated group were dosed with the IVP based on the dog’s body weight. Dogs weighing 4.0–22.0 lb (1.8–10 kg) received the 7.5 mg tablet and those dogs weighing more than > 22.1 lb (10 kg) received the 37.5 mg tablet. Dogs assigned to the control group were sham treated to maintain masking and to establish a time for post-treatment activities. After treatment on Day 0, dogs from treated and control groups were combed to determine the total live flea counts at the assigned time and analyzed as stated earlier.

### Target animal safety

The purpose of this GLP study was to assess the safety of the advantus™ soft chewable tablets when administered orally to Beagle puppies, starting at approximately 10 weeks old (±3 days), daily for six months at approximately 1, 3, and 5 times the targeted maximum exposure of 3.75 mg/kg body weight (the highest dose within the weight band).

Three treatment groups of four male and four female Beagle dogs were administered the advantus™ soft chewable tablets at the respective dose level of approximately 1, 3, or 5 times the daily dose once a day for 182 consecutive days. One additional group of eight (four animals/sex) served as the control and was sham dosed once a day for 182 consecutive days.

Observations for morbidity, mortality, injury and availability of food and water were conducted twice daily for all animals. Clinical observations were conducted twice per day, at least six hours apart (once in the AM and once in the PM). Body weights were measured and recorded three times per week during the acclimation period, three times during Week 1, twice during Week 2 and weekly thereafter. Dry and wet food consumption were measured, recorded daily and reported weekly during the dosing period. The individual daily food consumption was also reported. Ophthalmoscopic examinations were conducted pre-test and prior to the terminal necropsy. Physical and neurologic examinations were conducted pre-test, weekly for the first 4 weeks, and monthly thereafter. Blood and urine samples for clinical pathology evaluations were collected from all animals on Day −4 and at 1, 3, 4.5, and 6 months. Fecal samples were collected on Day −4 and at 1, 3, 4.5, and 6 months and were observed for color and consistency. Fecal samples were collected and direct and indirect ova and parasite evaluations were performed on all animals on Days −12 and −4. Blood samples for thyroid hormone (T4) evaluations were collected on Day −4 and at 1, 3, 4.5, and 6 months. At study termination, necropsy examinations were performed, organ weights were recorded, and tissues were microscopically examined.

The continuous endpoints measured during the study were analyzed by a repeated measures analysis of covariance with treatment, sex, day, treatment-by-sex, treatment-by-day, sex-by-day and treatment-sex-day terms in the model as fixed effects. Baseline measurements were included as covariates. Continuous endpoints measured once were analyzed by analysis of variance. All statistical comparisons of main effects and two-way interactions were performed at the 0.10 level of significance. The three-way interactions were performed at the 0.05 level of significance. Follow-up pairwise mean comparisons between the zero dose group and each non-zero dose group were performed, as necessary, using linear contrasts with significance level of 0.10.

### Field safety and efficacy

The objective of this GCP study in client owned dogs was to evaluate the field safety and effectiveness of daily oral treatment administered for 14 consecutive days, for the treatment of flea infestations on dogs. The study design was a masked placebo controlled multi-center field study conducted at various veterinary clinics. The individual household was the experimental unit and each household was randomly assigned to one of two treatment groups: daily oral IVP or placebo control (CVP). The numbers of cases enrolled in each group were in a ratio of 2:1. All personnel at each clinic were masked to the treatment groups except the person responsible for assigning the household to the treatment group. This person did not participate in any other study related activity. Both IVP and CVP soft chewable tablets were available in the same size and were indistinguishable from each other. The tablet bottles were labelled with letter codes, which were not available to the clinic personnel.

At each site, the investigator was the clinic veterinarian who was responsible for the protocol, oversight of owner communication, animal examinations and supervision of flea counts. The investigator ensured that the owners had signed an owner’s consent, had agreed to follow protocol requirements, and agreed to administer the daily treatments as described below. The owners had also demonstrated a clear understanding of the requirements of study participation and agreed to comply with study instructions, restrictions and visits.

Dogs enrolled in the study were 8 weeks of age or older, 4 pounds or greater in body weight and were healthy and in good condition. There were no breed or gender restrictions. No topical, systemic or environmental flea treatment or preventative products were allowed within the previous 30 days. Other concomitant medications that did not interfere with study results were allowed. These included mild conditions or disease that did not interfere with flea infestations. To qualify for inclusion the dog was required to have an affiliation with the household. Dogs excluded from the study included those receiving monthly oral or topical heartworm preventatives that also contained a flea treatment or control medication. Households with cats were included in the study, however all cats were treated at the clinic within 2 days of dog enrollment using any approved monthly flea treatment and control product.

Households with one or more dogs with flea infestations were enrolled in the study. The owners brought all the dogs to the clinic for the initial visit and a physical examination was conducted on all the dogs. A comb count was conducted on the dogs and the first dog, which had 10 or more fleas, was classified as the primary dog. All remaining dogs in the household were considered as secondary dogs. The household did not qualify for enrollment if none of the dogs in the household had 10 or more fleas. All dogs in each household received the same IVP or CVP treatment.

At each site, the qualifying households were assigned to a treatment group according to a pre-assigned randomization generated by a SAS statistical package to either the IVP or the CVP soft chewable tablet group. The dose tested was a minimum dose of 0.75 mg/kg body weight available in two sizes; 7.5 mg soft chewable tablets for dogs of 4.0 − 22.0 lb (1.8 − 10.0 kg body weight) and 37.5 mg soft chewable tablets for dogs of 22.1 − 110.0 lb (10.0 − 50.0 kg body weight). The investigator instructed the owners on the methods to administer the oral treatments, once a day for 14 consecutive days and document the treatments administered and any observations in the dosing diary. The owners were instructed to monitor the dogs for 1 hour after dosing for vomiting, coughing, gagging, retching, drooling, salivation or any other adverse events and record these observations in the dosing diary. The owners were instructed to bring the dogs back for the final visit for flea counts and safety assessments.

Field safety assessments were done during the initial visit (Day 1) and the final visit (Day 14). On Day 1, physical examinations were conducted on all dogs in the household, but on Day 14, it was only conducted on the primary dog. Blood samples were collected for routine hematology (CBC) and serum chemistry variables and T4 analysis. Urine samples were collected for routine urinalysis including specific gravity and pH evaluations.

The mixed model analysis included the fixed effect of ‘Treatment’, the random effects of ‘Site’ and the interaction of ‘Treatment and Site’ (random effect). Hematology, serum chemistry and urinalysis were statistically evaluated via an analysis of covariance (ANCOVA) using SAS PROC MIXED with the pre-treatment value used as covariate. The model included terms for the effects ‘Treatment’ and ‘Site’ as well as the interaction ‘Treatment by Site’. The terms ‘Site’ and ‘Treatment by Site’ were treated as random effects in the model.

## Results

### Pharmacokinetics

**Fig. 1:**
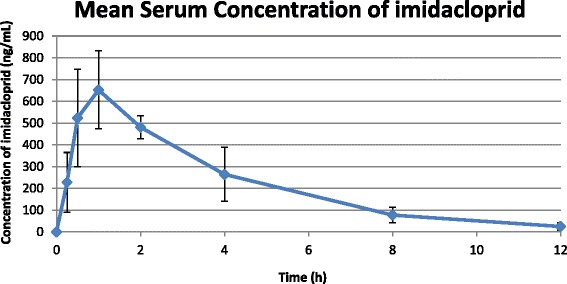
Serum concentration of imidacloprid following administration of advantus™ chewable tablets to dogs

 The body weight of the dogs ranged from 19.6 to 22.9 lb (8.9 kg to 10.4 kg ; mean 21.1 lb or 9.6 kg). Imidacloprid was identified in serum samples from all treated dogs with recoveries ranging from 82 % to 114 % with a mean of 104 % ± 9.7 %. Individual results for pharmacokinetic parameters, calculated using WinNonLin non-compartmental modeling for the variables T_max_, C_max_ and AUC_last_, are presented in Table 1 and a graph of the average serum concentration of imidacloprid (± SD) is shown in Fig. 1. The T_max_ was 1.31 hours, C_max_ was 690.0 ng/mL, AUC_last_ was 2615.5 h*ng/mL and the half-life was 2.2 hours following oral dosing of advantus™ soft chewable tablets.

**Table 1:** Summary of pharmacokinetic parameters in dogs following oral administration of advantus™ chewable tablets

Dog ID	T_max_ (h)	C_max_ (ng/mL)	AUC _last_ (h*ng/mL)	Half-life (h)
4153	1	654	3075.4	3.01
4181	1	557	1938.61	1.62
4374	1	764	2846.85	2.16
4386	0.5	693	2101.38	2.06
4140	4	512	3093.1	2.27
4175	1	812	2756.6	2.09
4182	1	777	2866.6	2.41
4383	1	751	2245.5	2.1
Mean	1.31	690	2615.5	2.2

### Dose confirmation

The 48 dogs enrolled in the dose confirmation study consisted of 34 Beagles, 1 Rat Terrier and 13 mixed breeds. There were 22 male and 26 females, who were 18.0 to 22.0 lb (8.18 − 10.0 kg ) in body weight, ranging from 9 months to 7.5 years in age. All dogs were susceptible to flea infestation as determined by the pre-treatment flea counts. Pre-treatment comb counts ranged from 75 to 100 fleas. These dogs were randomly allocated to treatment groups.

The animals were dosed with their assigned 7.5 mg IVP soft chewable tablet or the placebo CVP soft chewable tablet following confirmation of the dogs’ IDs (microchip reader or tattoos). The treatment administered ensured a minimum dose of 0.34 mg/lb BW (0.75 mg/kg BW). The actual dose administered to the dogs ranged from 0.34 – 0.42 mg/lb BW (0.75 − 0.92 mg/kg BW) of imidacloprid.

Dogs were combed until fleas were no longer found. Infestation of the three CVP groups were considered adequate as more than 50 % of fleas infesting the dogs were recovered at each combing period in more than six dogs per group. The mean flea counts recovered from each group and the calculated efficacy are listed in Table 2. The mean live flea counts from the IVP treated dogs were significantly lower than the counts from the CVP groups at each count time (p < 0.0001). The efficacy was 98.6 %, 99.9 % and 100 % at 8, 12 and 24 hours post-treatment, respectively. Therefore, advantus™ soft chewable tablets provided efficacy of >98 % at 8, 12 and 24 hours post-treatment at a minimum dose of 0.75 mg/kg body weight.

**Table 2:** Confirmation of 0.75 mg/kgbody weight as the dose of advantus™ chewable tablet

Time^1^	Treatment	Variable	N	Mean	Std Dev	Minimum	Maximum	Efficacy (%)	*p*-value
8	IVP^2^	Count	8	3.00	6.55	0.00	19.00		
		LogCount	8	0.31	0.46	0.00	1.30	98.63	<.00014
	CVP^3^	Count	8	85.13	27.37	18.00	100.00		
		LogCount	8	1.89	0.25	1.28	2.00		
12	IVP	Count	8	0.13	0.35	0.00	1.00	99.90	<.00014
		LogCount	8	0.04	0.11	0.00	0.30		
	CVP	Count	8	89.75	7.32	78.00	98.00		
		LogCount	8	1.96	0.04	1.90	2.00		
24	IVP	Count	8	0.00	0.00	0.00	0.00	100.00	<.00014
		LogCount	8	0.00	0.00	0.00	0.00		
	CVP	Count	8	91.13	6.62	82.00	100.00		
		LogCount	8	1.96	0.03	1.92	2.00		

^1^Time in hours post-treatment when efficacy was determined

^2^IVP is the advantus™ chewable tablets as the investigational veterinary product

^3^CVP is the placebo chewable tablets as control veterinary product

^4^Statistically significant at *p* < 0.01

### Knockdown and speed of kill

The 48 dogs enrolled in the knockdown and speed of kill study consisted of 41 Beagles and 7 mixed breeds. There were 26 males and 22 females, who were 14.3 to 37.1 lb (6.5 − 16.86 kg) in body weight and ranged from 8.2 months to 7.8 years in age. All dogs were susceptible to flea infestation as determined by the pre-treatment flea counts. Pre-treatment comb counts ranged from 45–100 fleas/dog. These dogs were randomly allocated to treatment groups.

The dogs were dosed with their assigned imidacloprid soft chewable tablet of either 7.5 or 37.5 mg. The dogs in the control group remained non-treated; however, a time was assigned to each dog to maintain masking and to establish a time for post-treatment activities. The identity of each dog was confirmed before each treatment was administered. The treatment administered ensured a minimum dose of 0.34 mg/lb (0.75 mg/kg) and the actual dose administered to the dogs ranged from 0.34 − 1.70 mg/lb (0.75 − 3.75 mg/kg) of imidacloprid. No adverse events were observed in any dog during the study.

All dogs were combed to conduct a complete body flea comb count to recover and count all live fleas. Dogs were combed until fleas were no longer found. Infestation of the control groups were considered adequate as more than 50 % of fleas infesting the dogs were recovered at each combing time point. The mean flea counts recovered from each group and the calculated efficacy are listed in Table 3. The mean live flea counts from the treated dogs were significantly lower than the counts from the control groups (*p* < 0.007) at 1, 4 and 24 hours post-treatment.

**Table 3:** Knockdown and Speed of kill efficacy of advantus™ chewable tablet administered at 0.75 mg/kg BW

Time^1^	Treatment	Variable	N	Mean	Std Dev	Minimum	Maximum	Efficacy(%)	*p*-value
0.5	Treated	Count	6	79.00	29.37	21.00	100.00	24.6	0.2719
		LogCount	6	1.86	0.25	1.34	2.00		
	Control	Count	6	94.33	4.63	88.00	100.00		
		LogCount	6	1.98	0.02	1.95	2.00		
1	Treated	Count	6	64.33	12.99	51.00	85.00	29.7	0.0073^2^
		LogCount	6	1.81	0.08	1.72	1.93		
	Control	Count	6	91.00	13.56	65.00	100.00		
		LogCount	6	1.96	0.07	1.82	2.00		
4	Treated	Count	6	3.17	1.60	2.00	6.00	96.6	<0.0001^2^
		LogCount	6	0.60	0.15	0.48	0.85		
	Control	Count	6	85.83	7.22	77.00	95.00		
		LogCount	6	1.94	0.04	1.89	1.98		
24	Treated	Count	6	0.17	0.41	0.00	1.00	99.9	<0.0001^2^
		LogCount	6	0.05	0.12	0.00	0.30		
	Control	Count	6	94.83	5.27	85.00	99.00		
		LogCount	6	1.98	0.02	1.93	2.00		

^1^Time in hours post-treatment when efficacy was determined

^2^Statistically significant

Flea efficacy was 24.6 %, 29.7 %, 96.6 % and 99.9 % at 0.5, 1, 4 and 24 hours post-treatment, respectively. Although dead fleas were noted at 0.5 hour, the number of live fleas recovered from the treated group and the control group was significantly different at 1 hour for the start of kill effect. The speed of kill at 4 hours post-treatment was > 90 %. The treatment was well tolerated and no abnormal clinical observations were observed in dogs when administered at a minimum dose of 0.75 mg/kg body weight.

### Target animal safety

Based on body weights and dose administered, the daily treatments administered were 3.74 mg/kg/day, 11.22 mg/kg/day and 18.7 mg/kg/day (0.9X, 2.7X and 4.5X). No effect of treatment was observed on any parameter evaluated including survival, physical examinations, bodyweights, food consumption, clinical pathology, clinical chemistry, urinalysis, fecal examinations, thyroid hormone (T4), and necropsy or histopathology examinations. The treatment was well tolerated with no evidence of toxicity observed in any of the parameters evaluated.

### Field safety and efficacy

Sixteen veterinary clinics at different geographical locations across the United States were recruited to conduct the study. Two hundred primary cases were enrolled of which 118 received the IVP soft chewable tablet and 82 received the CVP. A total of 131 secondary cases were enrolled of which 88 dogs received the IVP and 43 dogs received the CVP. Therefore, a total of 206 dogs were treated with IVP and 125 were treated with CVP. All treated dogs were included for safety evaluations but only 104 treated dogs and 69 control dogs were included for pivotal effectiveness evaluations. Several cases were excluded from pivotal effectiveness evaluation for various reasons including failure to complete the study, failure of the owners to return for the final visit, owners’ non-compliance with instructions, incorrect dosing or failure of the clinic to enroll the minimum required number of dogs.

Various breeds were enrolled in the study; the most common was mixed breed dogs. Up to 58 different breeds were recorded for the dogs enrolled in the study. The age of the dogs enrolled ranged from 2 months to 15 years. A total of 167 female and 164 male dogs were enrolled in the study. Of these dogs 109 females and 96 males were spayed or neutered. The weights of the dogs ranged from 4–118 lb (1.8 – 54 kg) and were administered 0.66 mg to 4.0 mg imidacloprid/kg body weight.

A total of 173 cases (104 dogs in the treated group and 69 dogs in the control group) were included in the pivotal effectiveness evaluation. The geometric mean live flea counts on Day 14 were 0.4 in the treated group and 21.0 in the control group. These counts were significantly different (*p* < 0.0001). The efficacy of advantus™ soft chewable tablets administered for 14 consecutive days was 98.2 %.

A total of 331 cases (206 dogs in the treated group and 125 dogs in the control group) were evaluated for field safety. No serious adverse events related to the IVP were reported during the study. Dogs in both groups had minor adverse events and the nature and frequencies of the adverse events observed are provided in Table 4. One dog had an episode of ataxia observed only on Day 7 and not reproduced on any other day. The dog was admitted to the hospital for further observation and no other clinical signs were observed.

**Table 4:** Summary of adverse events observed during the advantus™ soft chewable tablets field study

Adverse event	No. of cases in the IVP^1^ treated group	No. of cases in the CVP^2^ treated group
	(n (%) as total of 206 dogs)	(n (%) as total of 125 dogs)
Vomiting, upset stomach	8 (3.8 %)	4 (3.2 %)
Inappetence	6 (2.9 %)	3 (2.4 %)
Lethargy	4 (1.9 %)	4 (3.2 %)
Diarrhea, flatulence	3 (1.5 %)	4 (3.2 %)
Ataxia	1 (0.5 %)	None

^1^IVP is the advantus™ chewable tablets as the investigational veterinary product

^2^`CVP is the placebo soft chewable tablets as control veterinary product

Blood, serum, and urine samples were obtained from the primary dogs on Days 1 and on Day 14. An analysis of variance was performed on the variables to determine the difference between the treated versus the control groups. For the hematology analysis, although the means of all variables were within the normal range, the variables hematocrit, hemoglobin, MCHC and RBC were significantly higher in the treated group than the control group. These variables indicate loss of blood and were predicted because of the continued flea infestations on the control group. For serum chemistry, including T4 analysis, although the means of all variables were within the normal range, the variables that were significantly different were albumin, AST, calcium, cholesterol and total protein. The findings of albumin, calcium and total protein indicate loss of blood as a result of continued flea infestations of the control group. The difference in cholesterol was most likely due to the varied prandial states of the dogs at the time of serum collection, while the AST difference could be the result of concomitant use of corticosteroids in multiple dogs. There were no differences in any of the urinalysis variables.

Dosing naturally infested dogs with advantus™ soft chewable tablets for 14 consecutive days by dog owners was 98.2 % effective for the treatment of cat flea infestations. The mean live flea count in the treated group was significantly lower than the mean live flea count in the control group. No serious treatment related adverse events were observed in any dog during the study and it was concluded that advantus™ soft chewable tablets are safe for the treatment of flea infestation on dogs.

## Discussion

Neo-nicotinoids administered orally are characterized as products that have a rapid and complete absorption, resulting in rapid activity and a short half-life [24]. In this study, imidacloprid administered orally as soft chewable tablets demonstrated these characteristics. Imidacloprid was rapidly and completely absorbed with a T_max_ of approximately 1 hour and it was also rapidly eliminated with a half-life of approximately 2 hours. The C_max_ was approximately 700 ng/mL indicating that high levels were achieved within 1 hour, which would predict a rapid speed of kill activity.

Successful flea control requires understanding of individual factors of the flea biology and life cycle [25] that are playing out in each individual pet’s environment. Effective control depends on developing an integrated pest management program that is best suited for the individual client. Understanding sources of re-infestation is an integral part of this program. Fleas begin feeding as soon as they infest a host. A blood meal is required for egg production, which starts approximately 36 hours after feeding [26, 27]. Each flea may feed several times a day and may remain on the host for up to 21 days [26]. During this time, they produce many eggs that fall off the host and develop in the environment. Therefore, the majority of adult flea infestations are acquired as new infestations from the environment [27] and optimal control programs must be focused on eliminating the adult stages before they start laying eggs and seeding the environment. Quickly removing adult fleas infesting the dog is the first step in a successful flea control program. This can be achieved by using a product like advantus™ soft chewable tablets that are characterized by a fast onset of activity (start-to-kill) and a fast speed of kill. Oral imidacloprid treatment with advantus™ soft chewable tablets will eliminate flea infestations on the dog within 4 hours of treatment and prevent contamination of the environment with flea eggs.

Flea Allergy Dermatitis (FAD) is caused by flea bites in susceptible dogs and the feeding activity of one flea can initiate this reaction. In heavy infestations acute FAD can be an extremely debilitating and painful experience for the dog. Use of advantus™ soft chewable tablets administered to dogs with flea burdens will start to provide relief from biting fleas in one hour.

Fleas transmit several diseases to pets, through flea bites, flea feces or when fleas are ingested during grooming [3]. These diseases include, but are not limited to, bartonellosis (*Bartonella henselae*) and mycoplasmosis (*Mycoplasma haemofelis*). Fleas are also an intermediate hosts for *Dipylidium caninum* [28]. Quick removal of adult fleas using advantus™ soft chewable tablets (fast start-to-kill and a fast speed of kill) may aid in preventing flea-transmitted diseases in dogs.

A wide margin of safety of advantus™ soft chewable tablets was demonstrated with daily administration for 6 months at approximately 1, 3 and 5 times the recommend dose. No adverse events were reported which supports the extended daily use of the soft chewable tablets for dogs. Daily use of the soft chewable tablet will eliminate flea infestations on the dog before they start laying eggs. The presence of dogs in an infested environment will provide constant stimulation for the pre-adult stages of the fleas to emerge and infest the dog, but newly infesting fleas will be eliminated quickly by treatment of the dog with advantus™ soft chewable tablets. Studies have shown that in heavily contaminated environments it takes up to 2 months (60 days) to clean the environments of cycling flea populations [29].

## Conclusion

Development of advantus™ soft chewable tablets for the treatment of adult flea infestations of dogs was described. The characteristics of the product include convenience of administering imidacloprid as a soft chewable tablet that is rapidly absorbed within approximately 1 hour and eliminated rapidly as well, with a half-life of approximately 2 hours. These characteristics translate to rapid start to kill activity of 1 hour and speed of flea kill at 4 hours. Substantial evidence of efficacy was demonstrated with 96 % efficacy as early as 4 hours. The safety profile was excellent with no test substance related adverse events reported following 1, 3 and 5 times the dose administered daily for 6 months. Excellent safety and substantial efficacy was also demonstrated in a field efficacy study in client owned dogs.

## References

[1] Durden LA, Hinckle NC. Fleas (Siphonaptera). Mullen G and Durden LA, editors. Medical and Veterinary Entomology. Elsevier. 2009:p.115-135

[2] Conboy G (2009). Cestodes of dogs and cats. Vet Clin North Am Small Anim Pract.

[3] Little S, Starkey L. Conquering Fleas: Preventing infestations & limiting disease transmission. Today’s Vet Prac. 2012;Nov/Dec:33–38.

[4] Shaw SE, Day MJ, Birtles RJ, Breitschwerdt EB (2001). Tick-borne diseases of dogs. Trends Parasitol..

[5] Rust MK (2005). Advances in the control of *Ctenocephalides felis* (cat flea) on cats and dogs. Trends Parasitol..

[6] Arther RG, Cunningham J, Dorn H, Everett R, Herr LG, Hopkins T (1997). Efficacy of imidacloprid for the removal and control of fleas (*Ctenocephalides felis*) on Dogs. Am. J. Vet Res..

[7] Buckingham S, Lapied B, Corronc H, Sattelle F (1997). Imidacloprid actions on insect neuronal acetylcholine receptors. J Exp Biol..

[8] Dryden MW, Denenberg TM, Bunch S (2000). Control of fleas on naturally infested dogs and cats and in private residences using topical spot applications of fipronil or imidacloprid. Vet Parasitol..

[9] Everett R, Cunningham J, Arther R, Bledsoe DL, Mencke N (2000). Comparative evaluation of the speed of flea kills of imidacloprid and selamectin on dogs. Vet Ther..

[10] Matsuda K, Shimomura M, Kondo Y, Ihara M, Hashigami K, Yoshida N (2000). Role loop D of the α7 nicotinic acetylcholine receptor in its interaction with the insecticide imidacloprid and related neonicotinoids. British J Pharmacol..

[11] Arther RG, Cunningham J, Everett R (1997). Evaluating the effects of shampooing or repeated water exposure on the residual efficacy of Advantage® (imidacloprid) for flea control on dogs.

[12] Cunningham J, Everett R, Arther RG (1997). Effects of shampooing or water exposure on the initial and residual efficacy of imidacloprid. Suppl Comp Cont Educ Pract Vet..

[13] Young DR, Arther RG, Davis WL (2003). Evaluation of K9 Advantix™ vs. Frontline Plus® topical treatments to repel Brown Dog Ticks (*Rhipicephalus sanguineus*) on dogs. Parasitol Res.

[14] Epe C, Coati N, Stanneck D (2003). Efficacy of the compound preparation imidacloprid 10 % (w/v) / permethrin 50 % (w/v) spot-on against ticks (*I. ricinus*, *R. sanguineus*) and fleas (*C. felis*) on dogs. Parasitol Res.

[15] Mencke N, Volf P, Volfova V, Stanneck D (2003). Repellent efficacy of a combination containing imidacloprid and permethrin against sand flies (*Phlebotomus papatasi*) on dogs. Parasitol Res..

[16] Arther RG, Bowman DD, Slone RL, Travis LE (2005). Imidacloprid plus moxidectin topical solution for the prevention of heartworm disease (*Dirofilaria immitis*) in dogs. Parasitol Res..

[17] Von Samson-Himmelstjerna G, Epe C, Schimmel A, Heine J (2003). Larvicidal and persistent efficacy of an imidacloprid and moxidectin topical formulation against endoparasites in cats and dogs. Parasitol Res..

[18] Krieger K, Heine J, Dumont P, Hellmann K (2005). Efficacy and safety of imidacloprid 10 % plus moxidectin 2.5 % spot-on in the treatment of sarcoptic mange and otoacariosis in dogs: results of a European field study. Parasitol Res.

[19] Heine J, Krieger K, Dumont P, Hellmann K (2005). Evaluation of the efficacy and safety of imidacloprid 10 % plus moxidectin 2.5 % spot-on in the treatment of generalized demodicosis in dogs: results of a European field study. Parasitol Res.

[20] Fourie LJ, Kok DJ, Heine J. Evaluation of the efficacy of an imidacloprid 10 %/moxidectin1 % spot-on against *Otodectes cynotis* in cats. Parasitol Res. 2003;90:S112–3. 10.1007/s00436-003-0906-6 12928871

[21] Ross DH, Arther RG, von Simson C, Doyle V, Dryden MW (2012). Evaluation of the efficacy of topically administered imidacloprid + pyriproxyfen and orally administered spinosad against cat fleas (*Ctenocephalides felis*): Impact of treated dogs on flea life stages in a simulated home environment. Parasit Vectors..

[22] Marchiondo AA, Holdsworth PA, Fourie LJ, Rugg D, Hellmann K, Snyder DE (2013). World Association for the Advancement of Veterinary Parasitology (W.A.A.V.P.) second edition: guidelines for evaluating the efficacy of parasiticides for the treatment, prevention and control of flea and tick infestations on dogs and cats. Vet Parasitol.

[23] Veterinary International Conference on Harmonization (VICH) Guideline 43. Target Animal Safety for Veterinary Pharmaceutical Products, Step 4 draft, December, 2006.

[24] Kramer F, Mencke N (2001). Flea biology and control: The biology of cat flea, control and prevention with imidacloprid in small animals.

[25] Rust MK, Dryden MW (1997). The biology, ecology and management of the cat flea. Annu Rev Entomol..

[26] Halos L, Beugnet F, Cardoso L, Farkas R, Franc M, Guillot J (2014). Flea control failure? Myths and realities. Trends Parasitol.

[27] Dryden MW, Rust MK (1994). The cat flea: biology, ecology and control. Vet Parasitol..

[28] Eisen RJ, Gage KL (2012). Transmission of flea-borne zoonotic agents. Annu Rev Entomol..

[29] Dryden MW, Payne PA, Vicki S, Riggs B, Davenport J, Kobuszewski D (2011). Efficacy of dinotefuran-pyriproxyfen, dinotefuran-pyriproxyfen-permethrin and fipronil-(S)-methoprene topical spot-on formulations to control flea populations in naturally infested pets and private residences in Tampa. FL. Vet Parasitol..

